# Exploring Critical Eye-Tracking Metrics for Identifying Cognitive Strategies in Raven’s Advanced Progressive Matrices: A Data-Driven Perspective

**DOI:** 10.3390/jintelligence13020014

**Published:** 2025-01-26

**Authors:** Yaohui Liu, Keren He, Kaiwen Man, Peida Zhan

**Affiliations:** 1 School of Psychology, Zhejiang Normal University, Jinhua 321004, China; 2 Evaluation and Development Department of Henan Tianxing Education Technology Co., Ltd., Zhengzhou 450001, China; 3 Mental Health Education and Development Center, Zhejiang Normal University, Jinhua 321004, China; 4 Educational Studies in Psychology, Research Methodology, and Counseling, University of Alabama, Tuscaloosa, AL 35487, USA

**Keywords:** cognitive strategy, intelligence, matrix reasoning, eye movement, random forest

## Abstract

The present study utilized a recursive feature elimination approach in conjunction with a random forest algorithm to assess the efficacy of various features in predicting cognitive strategy usage in Raven’s Advanced Progressive Matrices. In addition to item response accuracy (RA) and response time (RT), five key eye-tracking metrics were examined: proportional time on matrix (PTM), latency to first toggle (LFT), rate of latency to first toggle (RLT), number of toggles (NOT), and rate of toggling (ROT). The results indicated that PTM, RLT, and LFT were the three most critical features, with PTM emerging as the most significant predictor of cognitive strategy usage, followed by RLT and LFT. Clustering analysis of these optimal features validated their utility in effectively distinguishing cognitive strategies. The study’s findings underscore the potential of specific eye-tracking metrics as objective indicators of cognitive processing while providing a data-driven method to identify strategies used in complex reasoning tasks.

## 1. Introduction

**Figure 1:**
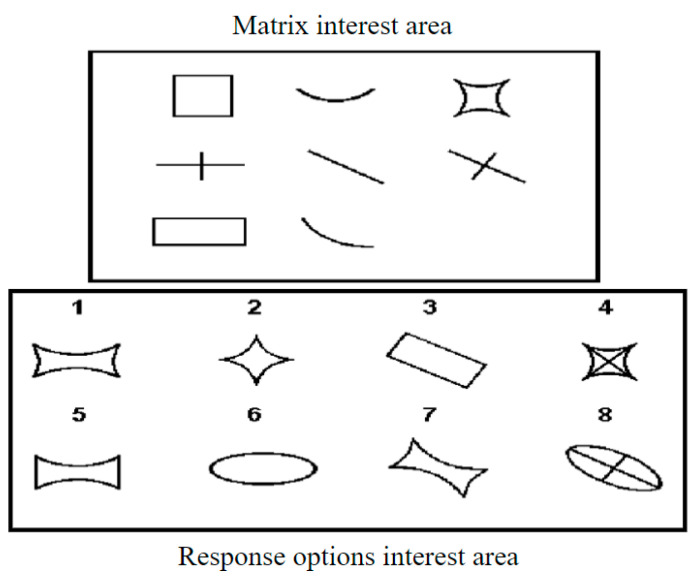
Example of an item in Raven’s advanced progressive matrices. This item involves a three-by-three matrix, with graphical elements in the matrix interest area and eight options in the response options area. One cell in the matrix is missing, and it needs to be selected from the response options area using analogy and inductive reasoning.

Raven’s Advanced Progressive Matrices (APM, Raven et al. 1998) is a widely utilized reasoning test in psychology, serving as a benchmark for gauging individuals’ overall intelligence (Loesche 2020). It consists of non-verbal multiple-choice items, each featuring a sequence of geometric patterns with one missing element, which the test taker is required to identify from a set of options. This format is designed to minimize cultural and linguistic biases, making it an effective tool for cross-cultural studies and assessments (Loesche 2020), as shown in Figure 1. Through a thorough examination of individuals’ performance on the APM, researchers gain insights into the developmental trajectories of cognitive processes, cognitive strategies, and reasoning abilities (Carpenter et al. 1990; Hayes et al. 2015; Vigneau et al. 2006; Niebaum and Munakata 2023). Additionally, the APM plays a pivotal role in clinical psychology, contributing significantly to the evaluation of diagnoses and treatment plans for disorders such as cognitive dysfunction and intellectual disability (Sukantarat et al. 2005; Vakil and Lifshitz-Zehavi 2012). The multifaceted applications of APM have spurred extensive research efforts, establishing it as a focal point in psychological inquiry.

The importance of researching cognitive strategies in the context of the APM cannot be overstated, as cognitive strategies reflect the thought processes and methods individuals employ when responding to APM tasks. Understanding these strategies offers valuable insights into fundamental cognitive processes and serves as a reference for the development of educational and cognitive training programs. Studies have shown that intervening in students’ cognitive strategies helps them think more effectively and perform better in tasks (Hessels et al. 2011; Hayes et al. 2015). Meanwhile, the early studies (Bethell-Fox et al. 1984; Vigneau et al. 2006) analyzing participants’ eye-tracking data have revealed that different individuals display distinct eye movement patterns when solving APM items, corresponding to different cognitive strategies. Examples include the constructive matching strategy and the response elimination strategy (Vigneau et al. 2006; Hayes et al. 2011). The constructive matching strategy involves forming a mental representation of the correct answer, while the response elimination strategy involves ruling out incorrect options through multiple comparisons of matrix elements and options (Vigneau et al. 2006).

Moreover, the use of these cognitive strategies is closely related to individual intelligence, working memory, item difficulty, and can significantly impact response accuracy (Gonthier and Roulin 2020; Li et al. 2022; Liu et al. 2023). For instance, individuals with higher intelligence tend to use the constructive matching strategy, and on certain items, those using the constructive matching strategy are more likely to answer correctly (Liu et al. 2023). In-depth research on these cognitive strategies has greatly advanced our understanding of individual cognitive processes and intellectual development, shedding light on the thinking processes of different groups and providing an important theoretical and empirical foundation for cognitive psychology and intelligence research (Laurence and Macedo 2023). In addition, cognitive strategy research plays a key role in refining the design and assessment of reasoning tests, improving their accuracy and validity (Becker et al. 2016; Arendasy and Sommer 2013).

Since cognitive strategies occur in the brain, they are not easily observed, and the identification of cognitive strategies is the foundation work for conducting research on cognitive strategies. So, the identification of cognitive strategies in APM has been a focal point of psychological research, leading to the development of various methodologies. Current methods for identifying cognitive strategies can be categorized into four main types: questionnaire-based methods, think-aloud protocols, eye-tracking data analysis, and mouse-tracking data analysis (Laurence and Macedo 2023; Liu et al. 2023). The first two methods, while insightful, have notable limitations. Self-report questionnaires, for example, rely heavily on participants’ introspection and memory, which can introduce biases and inaccuracies (Jarosz et al. 2019). Think-aloud protocols, in which participants verbalize their thought processes while solving problems, provide rich qualitative data but are time-consuming and may alter the natural problem-solving process due to the dual task of thinking and verbalizing (Chiu and Shu 2010; Laurence and Macedo 2023).

Eye-tracking data analysis has emerged as a powerful tool for understanding cognitive strategies due to the objective and non-intrusive nature of eye-tracking collection. By recording and analyzing eye movements, researchers can infer the strategies used by individuals during tasks. For example, eye-tracking metrics, such as fixation duration and the number of toggles between different areas of interest, provide insights into whether a participant is using a constructive matching strategy or a response elimination strategy (Vigneau et al. 2006). Hayes et al. (2011) and Liu et al. (2023) developed different methods for identifying strategies from data-driven and theory-driven perspectives, respectively. Despite its advantages, this method also faces challenges. A significant disadvantage is the complexity of the methods and model interpretation. For example, advanced methods, such as Markov models and transfer matrices, as proposed by Hayes et al. (2011), require complex analytical tools and expertise. Similarly, the parameter estimation involved in the multi-strategy eye-tracking model (MEM) proposed by Liu et al. (2023) requires a certain programming foundation, which may limit their wider application in research.

Mouse-tracking data analysis captures the movement and clicks of a computer mouse as participants interact with tasks (Rivollier et al. 2021). This method provides a less intrusive and more cost-effective alternative to eye-tracking. Researchers need to develop specific programs to use mouse movements to simulate the process of eye movements to obtain information. For example, positioning the mouse over the response options area reveals this section while the matrix area disappears, simulating a gaze focused on the option to gather information. By analyzing mouse movement patterns or indicators (e.g., the amount of time the mouse stays in the response options interest area), researchers can infer cognitive strategies similar to those identified through eye-tracking (Rivollier et al. 2021). However, this method has notable limitations. Unlike eye movements, mouse movements are intentional actions rather than subconscious processes, potentially falling short in capturing the spontaneous cognitive processes involved in strategy use. Additionally, the structure of the test may be altered in such setups, potentially impacting judgments about individuals’ strategy use (Laurence and Macedo 2023).

Overall, compared to other methods, the collection of eye-tracking data does not interfere with the individual’s response process, allowing for a more direct reflection of their thought process during task performance. Furthermore, the richness and comprehensiveness of eye-tracking data provide significant potential for inferring cognitive strategies.

Different eye-tracking metrics are used to infer the use of strategies in different studies, and various studies highlight different eye-tracking metrics as effective predictors of cognitive strategy usages. For example, Vigneau et al. (2006) identified latency to first toggle (LFT), proportional time-on matrix (PTM), and number of toggles (NOT) as useful metrics for predicting strategy use. A higher NOT, shorter LFT, and smaller PTM correspond to the response elimination strategy, while a lower NOT, longer LFT, and larger PTM indicate the constructive matching strategy. In Laurence et al.’s (2018) study, the rate of toggling (ROT) was considered the best predictor of strategy use, correlating with better performance. Raden and Jarosz (2022) also affirmed the importance of the ROT in predicting strategy use when studying participants’ strategy use across reasoning tasks. In contrast, Liu et al. (2023) found that PTM and rate of latency to first toggle (RLT) are effective metrics for predicting strategy use, but ROT is not. These differences highlight an ongoing challenge in the field: the lack of consensus on which eye-tracking metrics are the most reliable predictors of cognitive strategies. This variability can stem from differences in the metrics used or the analytical methods applied across studies. As a result, there is a pressing need for standardized methodologies and comparative studies to establish more consistent and generalizable findings. Additionally, some studies (Gonthier and Roulin 2020; Laurence et al. 2018; Gonthier and Thomassin 2015; Liu et al. 2023) have suggested that strategy affects response accuracy as well as response time. Therefore, whether response accuracy (RA) and response times (RT) can be used to predict strategy use alongside eye-tracking metrics is an important question.

The present study aims to explore which features (i.e., RA, RT, and the five eye-tracking metrics [PTM, ROT, LFT, NOT, RLT]) are most effective for predicting cognitive strategies by employing a recursive feature elimination approach in conjunction with a random forest model. Subsequently, the selected features will be used for clustering analysis to investigate whether the classification results align with theoretical cognitive strategies and to assess the validity of the cluster analysis in identifying strategies using the selected features.

The remaining sections review relevant background information, including the three cognitive strategies involved in APM and the MEM, a strategic measurement model used to distinguish strategies and label data for the random forest model, which is a supervised machine learning method. An empirical study was then conducted to identify the subset of features that are most valuable for predicting strategies. This was followed by a cluster analysis of the selected features and a detailed description of the clustering results. Finally, a summary of the findings and a discussion of future research directions are presented.

## 2. Backgrounds

### 2.1. Three Cognitive Strategies in APM

Initially, previous studies identified two common cognitive strategies employed in APM: constructive matching and response elimination (Bethell-Fox et al. 1984; Snow 1980; Vigneau et al. 2006). Constructive matching involves participants extracting complete answer rules from the matrix area, mentally constructing the final answer, and making a selection from the options area. In contrast, response elimination entails participants extracting only part of the answer rules from the matrix area, gradually eliminating options that do not fit based on these partial rules and arriving at the final answer.

In recent years, Jarosz et al. (2019) proposed the possibility of another cognitive strategy—the isolate-and-eliminate strategy—which can be considered a hybrid of constructive matching and response elimination. In this strategy, individuals identify specific rules governing the problem, eliminate incorrect options based on these rules, and then refine their choices by isolating the most probable correct answer. Liu et al. (2023) also observed the mixed use of constructive matching and response elimination strategies, supporting Jarosz et al.’s (2019) argument.

### 2.2. Five Eye-Tracking Metrics

In analyzing cognitive strategies using eye-tracking data, several key eye-tracking metrics have been identified. These metrics offer valuable insights into the processes individuals use to solve matrix reasoning tasks. Below are five important eye-tracking metrics, along with their definitions, meanings, calculation methods, and their relationship to cognitive strategies.

#### 2.2.1. Proportional Time on Matrix (PTM)

PTM is a measure of the proportion of time a participant spends looking at the matrix area (*T^matrix^*^)^ relative to the total time spent on the item (*T^item^*). It is calculated using the formula: *PTM* = *T^matrix^*/*T^item^*. This metric is important because participants employing the constructive matching strategy typically spend a high proportion of their gaze time on the matrix area, as they form a mental representation of the correct answer before making a choice (Vigneau et al. 2006). A higher PTM indicates deeper engagement with the problem matrix, which is characteristic of the constructive matching strategy.

#### 2.2.2. Latency to First Toggle (LFT)

LFT measures the time taken from the start of the task until the participant first shifts their gaze to the options area. This metric is calculated as the time elapsed from the beginning of the task to the first toggle. A longer LFT suggests that the participant is spending more initial time analyzing the matrix, indicative of the constructive matching strategy. Conversely, a shorter LFT reflects a quicker shift to the options area, which aligns with the response elimination strategy (Vigneau et al. 2006).

#### 2.2.3. Rate of Latency to First Toggle (RLT)

RLT is the ratio of the latency to the first toggle to the total time spent on the item. It is calculated using the formula: *RLT* = *LFT*/*T^item^*. This metric indicates the proportion of the initial time spent on the matrix before considering the options. Participants using the constructive matching strategy typically have a higher RLT, as they invest more time in understanding the problem rules and extracting information from the matrix area before looking at the options (Liu et al. 2023).

#### 2.2.4. Number of Toggles (NOT)

NOT counts the total number of gaze shifts between the matrix and the options area during the task. This metric is important because a higher number of toggles corresponds to the response elimination strategy, where participants frequently scan back and forth to eliminate incorrect options. In contrast, a lower number of toggles indicates a more focused and deliberate approach, characteristic of the constructive matching strategy (Vigneau et al. 2006; Hayes et al. 2011).

#### 2.2.5. Rate of Toggling (ROT)

ROT measures the frequency of toggles between the matrix and the options area per second. It is calculated as *ROT* = *NOT*/*T^item^*. Participants employing the response elimination strategy tend to have a higher rate of toggling, as they toggle more often within a given time. Conversely, participants using the constructive matching strategy exhibit a lower rate of toggling because they spend more time forming a comprehensive understanding of the problem before making a decision (Vigneau et al. 2006; Laurence et al. 2018).

Overall, these metrics—PTM, LFT, RLT, NOT, and ROT—provide valuable insights into the cognitive strategies individuals use during problem-solving tasks. By analyzing these eye-tracking data, researchers can identify more effective metrics and improve the accuracy of strategy prediction models.

### 2.3. Multi-Strategy Eye-Tracking Model

The MEM is a theory-driven psychometric model that is designed to estimate the probability of cognitive strategy usage by participants and their intelligence. In contrast to existing “black-or-white” approaches to strategy identification, the MEM estimates participants’ strategy use and further identifies it in the form of probabilities, which opens up the possibility of using MEM to discover a third strategy. Specifically, Liu et al. (2023) observed that for some specific items, some participants did not use specific cognitive strategies of construct matching and response elimination strategies with high probability, but rather showed ambiguity in strategy use, most likely by using both strategies (i.e., the isolate-and-eliminate strategy) in the problem-solving process.

For the APM, assuming *I* participants using *M* = 2 strategies (i.e., constructive matching and response elimination strategies) with *J* = 36 items, the MEM can be expressed as:

(1) $$P\left(Y_{ij}=1|\theta_{i}\right)=\sum_{m=1}^{M}P\left(Y_{ij}=1|\theta_{i},m_{ij}\right)\times \;P\left(m_{ij}\right)$$

where $P\left(Y_{ij}=1|\theta_{i},m_{ij}\right)$ represents the strategy implementation model, and $P\left(m_{ij}\right)$ represents the strategy selection model. Specifically, *m_ij_* = 1 indicates that participant *i* applied the constructive matching strategy to item *j*, and *m_ij_* = 2 indicates that participant *i* applied the response elimination strategy to item *j*.

The MEM hypothesis posited that participants utilizing the constructive matching strategy would exhibit a higher or equal probability of providing correct responses compared to those employing the response elimination strategy (Gonthier and Roulin 2020; Mitchum and Kelley 2010; Laurence and Macedo 2023), as follows:

(2) $$P\left(Y_{ij}=1|\theta_{i},m_{ij}=1\right)\geq P\left(Y_{ij}=1|\theta_{i},m_{ij}=2\right)$$

Specifically,

(3) $$P\left(Y_{ij}=1|\theta_{i},m_{ij}=1\right)=\frac{exp\left(\theta_{i}-b_{j}+e_{j}\right)}{1+exp\left(\theta_{i}-b_{j}+e_{j}\right)}$$

(4) $$P\left(Y_{ij}=1|\theta_{i},m_{ij}=2\right)=\frac{exp\left(\theta_{i}-b_{j}\right)}{1+exp\left(\theta_{i}-b_{j}\right)}$$

where *b_j_* denotes the difficulty of item *j*, *θ_i_* denotes the latent ability (i.e., intelligence) of participant *i*; *e_j_*, which in formula 3 is the strategy sensitivity parameter and is constrained to be non-negative (i.e., $e_{j}\geq 0$), representing the gain in the correct response probability by using the constructive matching strategy compared to using the response elimination strategy on item *j*.

In addition, the strategy selection model represents the probability of participant *i* applying strategy *m* on item *j*, and $\sum_{m=1}^{M}P\left(m_{ij}\right)=1$. It is inferred using three eye-tracking measures (i.e., PTM, ROT, and RLT), and its value is constrained to a number between 0 and 1, with a logistic function as follows:

(5) $$P\left(m_{ij}=1\right)=\frac{exp\left(\omega_{1}\times f_{1ij}+\omega_{2}\times f_{2ij}+\omega_{3}\times f_{3ij}\right)}{1+exp\left(\omega_{1}\times f_{1ij}+\omega_{2}\times f_{2ij}+\omega_{3}\times f_{3ij}\right)},$$

and

(6) $$P\left(m_{ij}=2\right)=1-P\left(m_{ij}=1\right),$$

where $P\left(m_{ij}=1\right)$ and $P\left(m_{ij}=2\right)$ represent the probability that participant *i* used the constructive matching strategy and the response elimination strategy, respectively, on item *j*; $f_{1ij}$, $f_{2ij}$, and $f_{3ij}$ represent participant *i*’s PTM, ROT, and RLT on item *j* (each eye-tracking metric for all participants is standardized for each item to put all weight parameters on the same scale), and $\omega_{1}$, $\omega_{2}$, and $\omega_{3}$ represent the weights of the three eye-tracking measures, indicating the degree to which the three eye-tracking measures influence the probability of strategy selection by the participant.

According to the MEM, strategy identification can be achieved using two approaches. The first one is based on whether $P\left(m_{ij}=1\right)$ is greater than or equal to a particular cut-off point (e.g., 0.5): when $P\left(m_{ij}=1\right)$ exceeds the cut-off point, the participant is identified as using the constructive matching strategy on item *j*, and when $P\left(m_{ij}=1\right)$ is less than one minus the cut-off point, the participant is identified as using the response elimination strategy on item *j*. Another one is based on whether the posterior probability distribution of $P\left(m_{ij}=1\right)$ is significantly different from 0.5. When the posterior probability distribution of $P\left(m_{ij}=1\right)$ is significantly greater than 0.5 (i.e., the 2.5% highest posterior density of $P\left(m_{ij}=1\right)$ exceeds 0.5), it is considered that the participant uses the constructive matching strategy on item *j*. Conversely, when the posterior probability distribution of $P\left(m_{ij}=1\right)$ is significantly less than 0.5 (i.e., the 97.5% highest posterior density of $P\left(m_{ij}=1\right)$ is less than 0.5), it is considered a response elimination strategy on item *j*. While, the remaining cases are determined to be an isolate-and-eliminate strategy, which is a combination of the two strategies (Liu et al. 2023).

### 2.4. Recursive Feature Elimination and Random Forest Algorithm

Recursive feature elimination is a commonly used feature selection method for regression and classification problems. Its basic idea is to remove features recursively in continuous iterations until a predefined number of features is reached (Darst et al. 2018).

**Figure 2:**
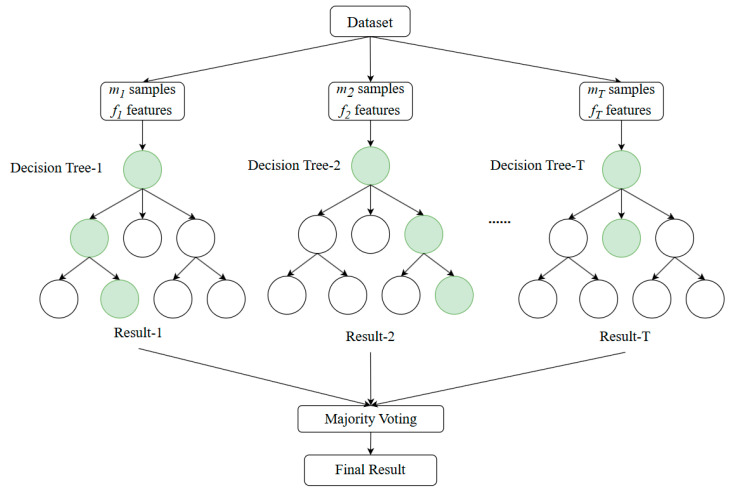
A brief schematic of the random forest algorithm. Each circle represents a node. Green means the path node with the highest probability value.

Random forest is an ensemble learning algorithm developed based on decision trees, meaning a random forest consists of multiple decision trees (Breiman 2001). By combining the predictions of multiple decision trees, the random forest algorithm can significantly improve the model’s accuracy and robustness (Biau and Scornet 2016). Each tree is independently generated during training. For trees *t* (*t* = 1, 2, …, *T*), random samples with replacement (*m* samples) and random subsets of features (*f* features) are drawn from the training set to construct each tree (Figure 2). This introduces diversity in the decision-making process of each tree.

In classification tasks, the random forest algorithm determines the final classification result by voting on the results of each tree. Due to its integration of multiple trees’ decisions, the random forest has strong resistance to overfitting and is less sensitive to noise and missing values, making it widely applicable in various fields (e.g., Han et al. 2019; Biau and Scornet 2016; Rigatti 2017).

Another significant advantage is that random forests can be used for feature importance evaluation. Mean decrease in impurity (MDI; Louppe et al. 2013), central to feature importance in Random Forest models, is a measure of each feature’s contribution to reducing misclassification. During model training, each time a feature is used to split a node, the impurity of that node decreases, as it divides the data into more homogeneous subsets. The total decrease in impurity is accumulated for each feature across all trees in the forest and averaged, yielding the MDI score for each feature. In general, higher MDI values indicate a more substantial contribution to classification accuracy, as these features play a more critical role in reducing misclassification. While there is not a strict cutoff value for MDI, it is mainly used to rank features relative to one another. Features with very low or near-zero MDI values may be candidates for elimination, as their impact on predictive accuracy is minimal. The formula for MDI can be expressed as:

(7) $$MDI\left(f\right)=\frac{1}{T}\sum_{t=1}^{T}\sum_{n\in Nodes\left(t\right)}\frac{N_{n}}{N}△I_{n}\left(f\right)$$

where *f* represents the feature, *T* is the total number of decision trees in the random forest, and *t* denotes the *t*-th decision tree. Notes (*t*) represent the set of all nodes in the *t*-th tree. *N_n_* is the number of samples in node *n* and *N* is the total number of samples in the entire dataset. Δ*I_n_*(*f*) represents the decrease in impurity at node *n* caused by feature *f*. This is the amount by which the impurity of the dataset decreases when the subset *N_n_* at node n is split into two subsets (*N_nL_*, *N_nR_*) based on feature *f*. The formula for Δ*I_n_*(*f*) can be expressed as:

(8) $$△I_{n}\left(f\right)=I\left(N_{n}\right)-\left(\frac{N_{nL}}{N_{n}}I\left(N_{nL}\right)+\frac{N_{nR}}{N_{n}}I\left(N_{nR}\right)\right)$$

where *I(N_n_)* is the impurity of the dataset with *N* samples at node *n, N_nL_* and *N_nR_* are the number of samples in the left and right subsets after the split, and *I(N_nL_)* and *I(N_nR_)* are the impurities of the left and right subsets, respectively. Furthermore, dataset impurity is commonly measured using the Gini index or Shannon entropy (Louppe et al. 2013). In the present study, The Gini index was chosen to represent the decrease in impurity (Lerman and Yitzhaki 1984). The Gini index for a sample set is calculated using the following formula:

(9) $$Gini\left(N\right)=1-\sum_{k=1}^{K}p_{k}^{2}$$

where *K* is the number of classes and *p_k_* is the proportion of samples belonging to class *k* in the dataset *N*.

## 3. Empirical Study

### 3.1. Data Description

For the present study, specific eye-tracking data from Liu et al. (2023) involving 192 college students on APM (36 items) were used due to its relatively large size in the field of reasoning studies combining eye-tracking, making it more representative. All participants were randomly selected from a university in a coastal province in China and had not participated in the APM before. The sample consisted of 147 females and 45 males, with an average age of 22.06 years (SD = 2.54) (socioeconomic status details were not included in the original dataset).

Each participant was instructed to complete the APM test individually on a computer. An EyeLink device (EyeLink Portable Duo, SR Research Ltd., Oakville, ON, Canada) was employed to record eye-tracking data during the test, while a chin rest was used to stabilize participants’ heads to minimize errors in the eye-tracking data caused by head movements. Participants were asked to first examine each item and then press the space bar to proceed with their response. A similar response procedure was adopted in the study conducted by Hayes et al. (2011). A more detailed description of this dataset, including the acquisition process, can be found in Liu et al. (2023). As aforementioned, seven features, including RA, RT, and five eye-tracking metrics (i.e., PTM, LFT, RLT, NOT, and ROT), were analyzed.

### 3.2. Analysis

Recursive feature elimination and the random forest model were used to assess the importance of seven features in the context of two-class classification (constructive matching and response elimination strategies) and three-class classification (constructive matching, response elimination, and isolate-and-eliminate strategies), and to select the optimal combination of features. The specific steps are as follows:

(1) Initialize the feature subset: An initial subset of *k* features was selected and input into a Random Forest model. Importance scores (i.e., MDI) for each feature were computed based on their contribution to reducing classification error across the ensemble of decision trees. To ensure a robust evaluation, 10-fold cross-validation was applied. In this method, the dataset was partitioned into ten equal parts (folds), with nine folds used for training and one for testing in each iteration, cycling through all ten folds. The average classification accuracy across these folds provided a reliable estimate of the performance of the initial feature subset.
(2) Remove the least important feature: After computing the importance scores, the feature with the lowest importance was removed from the subset, resulting in a new subset of *k* − 1 features. This reduced subset was re-input into the Random Forest model, where importance scores were recalculated for the remaining features. Another round of 10-fold cross-validation was then performed on the updated subset to obtain a new mean classification accuracy score, allowing for the evaluation of model performance as the feature count was gradually reduced.
(3) Perform the recursive process: Steps (1) and (2) were repeated iteratively, with the least important feature removed in each step until no features remained in the subset. This recursive elimination generated a sequence of k feature subsets, each containing progressively fewer features. For each subset, 10-fold cross-validation was performed, recording classification accuracy scores for all subsets to illustrate how model performance varied as features were removed. This systematic approach facilitated the identification of an optimal balance between model simplicity and predictive power.
(4) Select the optimal feature combination: Choose the feature subset with the highest classification accuracy as the optimal feature combination.

The identification results of the MEM were used as labeled data in the dataset. We used whether the posterior probability distribution of $P\left(m_{ij}=1\right)$ was significantly different from 0.5 to identify three cognitive strategies. Subsequently, the datasets were trained using random forest algorithms for both two-class and three-class classifications to evaluate which metrics are critical in predicting the use of cognitive strategies. For the three-class classification, the entire dataset was used for training. However, for the two-class classification, only data identified as corresponding to the constructive matching and response elimination strategies were employed.

Based on the aforementioned methods, after selecting the optimal subset of features for predicting strategies, K-means clustering analysis will be conducted using this feature subset to explore whether it is possible to categorize the data into two or three classes. This will help to verify the validity of the results regarding the importance of features in another way. The determination of an optimal number of clusters is investigated by comparing the silhouette coefficients’ magnitudes (Rousseeuw 1987) across different number of clusters (*K* = 2, 3, …, 10). Each case corresponds to an average silhouette coefficient. The silhouette coefficient of a data point *n* is calculated as: $sc\left(n\right)=\frac{b\left(n\right)-a\left(n\right)}{max\left(a\left(n\right),b\left(n\right)\right)}$, where *a*(*n*) is the average distance from a data point n to other data points in the same cluster, and *b*(*n*) is the average distance from the data point n to all data points of the nearest cluster. Finally, the silhouette coefficients of all data points are averaged to obtain the average silhouette coefficient of the clustering result. The silhouette coefficient takes the value between [−1, 1], and a larger value indicates large inter-class distances and small intra-class distances, which also means better classification. Subsequently, the features of different categories will be analyzed according to the optimal number of clusters.

The recursive feature elimination, random forest, and K-means algorithms were applied using the scikit-learn package (version 1.3.2; Pedregosa et al. 2011) in Python (version 3.9). Additionally, the MEM was implemented using the pyjags package (version 1.3.8; Plummer 2012) with settings identical to those in the study by Liu et al. (2023). To increase the repeatability of the current study, data are available at https://osf.io/rgt6q/?view_only=4df30b8d57da4ad8993d5a1279419e27 (accessed on 5 January 2025) and the analysis code used is available upon request from the corresponding author.

## 4. Result

**Figure 3:**
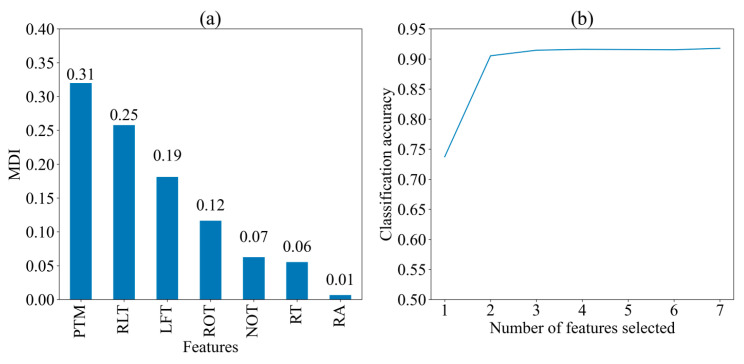
Importance of seven features for two cognitive strategies. (**a**) Mean decrease in impurity (MDI) of seven feature; (**b**) Classification Accuracy with Different Number of Features. PTM: proportional time on matrix; RLT: rate of latency to first toggle; LFT: latency to first toggle; ROT: rate of toggling; NOT: number of Toggles; RA: response accuracy; RT: response time.

Figure 3 displays the MDI of seven features and the classification accuracy of the random forest algorithm when different numbers of features are selected for predicting two strategies, respectively. The importance of the features is presented in descending order of MDI values: PTM (0.31), RLT (0.25), LFT (0.19), ROT (0.12), NOT (0.07), RT (0.06), and RA (0.01). This order indicates the significance of each feature in contributing to the model’s predictions. The classification accuracy exceeds 0.9 when two features (PTM and RLT) are selected, and it remains relatively stable as more than three features (PTM, RLT, and LFT). This indicates that PTM, RLT, and LFT are the most three critical features for prediction, while additional features contribute marginally to the model’s performance.

**Figure 4:**
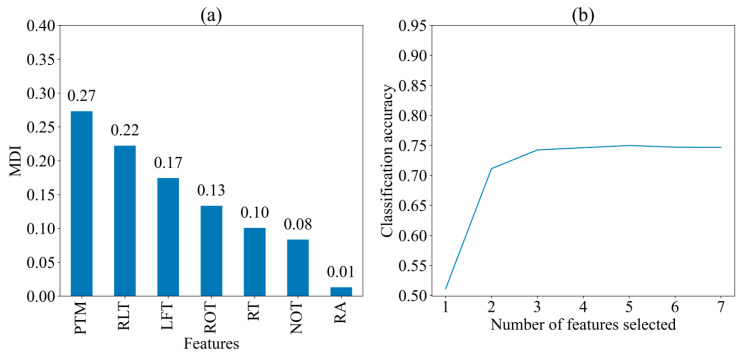
Importance of seven features for three cognitive strategies. (**a**) Mean decrease in impurity (MDI) of seven feature; (**b**) Classification Accuracy with Different Number of Features. PTM: proportional time on matrix; RLT: rate of latency to first toggle; LFT: latency to first toggle; ROT: rate of toggling; NOT: number of Toggles; RA: response accuracy; RT: response time.

Figure 4 displays the MDI of seven features and the classification accuracy of the model when different numbers of features are selected for predicting three strategies, respectively. The importance of the features was broadly consistent with that in the two cognitive strategies, presented in descending order of MDI values: PTM (0.27), RLT (0.22), LFT (0.17), ROT (0.13), RT (0.10), NOT (0.08), and RA (0.01). The classification accuracy exceeds 0.7 when two features (PTM and RLT) are selected for classification, and it remains relatively stable with more than three features (PTM, RLT, and LFT) are selected. This means that the three most important features and their ordering did not change when predicting the three strategies, despite the decrease in classification accuracy.

Overall, PTM, RLT, and LFT are consistently important in predicting cognitive strategies, whether considering two or three strategies, while other features play a minor role.

**Figure 5:**
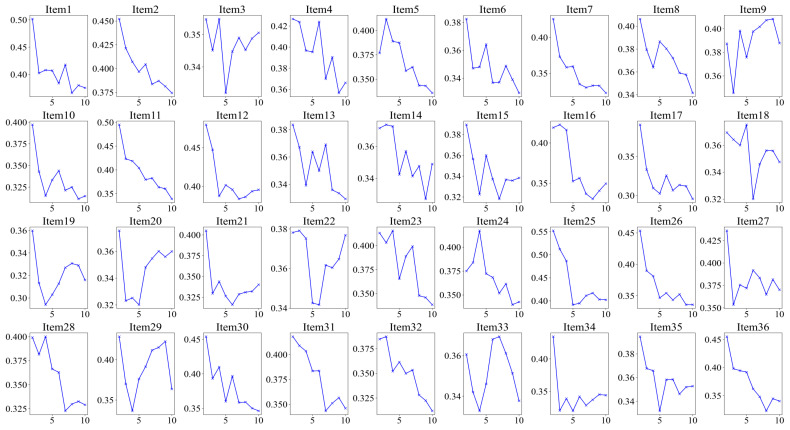
Silhouette coefficients on each item for K-means with 2~10 clusters for the three eye-tracking features. The highest value represents the optimal number of clusters.

**Figure 6:**
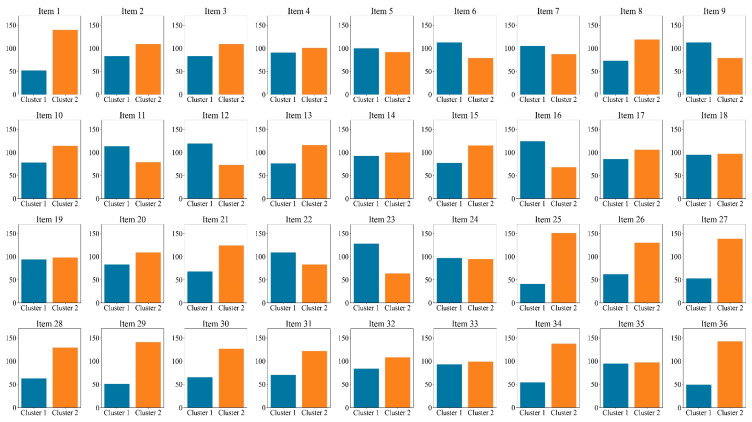
Number of participants in two clusters on each item.

**Figure 7:**
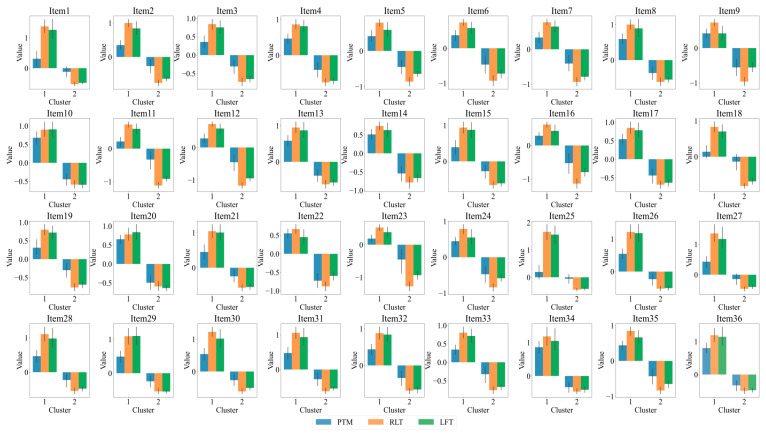
Histogram of three eye-tracking features on two clusters for each item. PTM: Proportional time on matrix; RLT: Rate of latency to first toggle; LFT: Latency to first toggle; Error bar is the 95% confidence interval of the overall mean.

**Figure 8:**
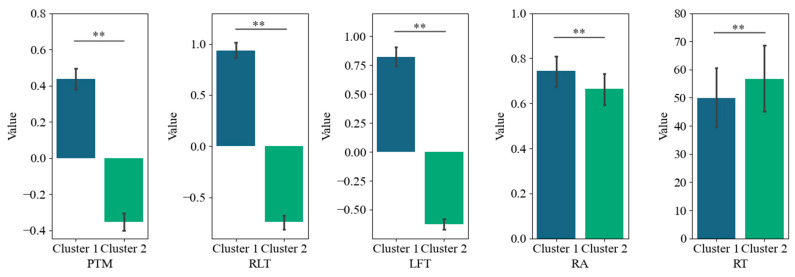
Paired-sample t-test of PTM, RLT, LFT, RA, and RT. PTM: Proportional time on matrix; RLT: Rate of latency to first toggle; LFT: Latency to first toggle; RA: response accuracy; RT: response time. Error bar is the 95% confidence interval of the overall mean. ** represents *p* < 0.01, sample N = 36.

Furthermore, we conducted a K-means cluster analysis of the three eye-tracking metrics, PTM, RLT, and LFT, in order to explore whether it is possible to categorize the data into two or three classes. Figure 5 displays the average silhouette coefficients for each item when clustered into clusters 2~10. The results showed that, for the majority of the items (27 items), the dataset was optimally categorized into two clusters, followed by four clusters (5 items), which means participants were optimally categorized into two clusters based on these three eye-tracking metrics. Figure 6 and Figure 7 display the number of participants in two clusters and three eye-tracking metrics on each item, respectively. The number of participants categorized as Cluster 1 was lower than that in Cluster 2 across the majority of items. And for each of the three eye-tracking metrics, the participants in Cluster 1 exhibited significantly higher values than those in Cluster 2. This suggests that participants in Cluster 1 spend more time in the matrix area before looking at the options area for the first time and also focus more attention on the matrix area overall than those in Cluster 2. This indicates that they tend to extract more information from the matrix. Meanwhile, the paired-samples t-test results, as illustrated in Figure 8, revealed that participants in Cluster 1 demonstrated significantly higher values on PTM (*t* = 19.45, *p* < 0.01, Cohen’s *d* = 5.41), RLT (*t* = 70.78, *p* < 0.01, Cohen’s *d* = 7.56), and LFT (*t* = 47.36, *p* < 0.01, Cohen’s *d* = 6.91) across the 36 items compared to participants in Cluster 2. Additionally, participants in Cluster 1 exhibited significantly greater response accuracy (*t* = 5.35, *p* < 0.01, Cohen’s *d* = 0.38) and shorter response times (*t* = 5.14, *p* < 0.01, Cohen’s *d* = 0.21) on these items relative to Cluster 2.

Based on the above results and the typical characteristics of the constructive matching and response elimination strategies, Cluster 1 and Cluster 2 can be inferred to represent the constructive matching strategy group and the response elimination strategy group, respectively. In such cases, the consistency between the clustering results and the identification results from the MEM was 82%. Further, PTM, RLT, and LFT were put into the MEM for strategy identification, and the results show an 82% consistency with the results of the study of Liu et al. (2023) and a 76% consistency with the results from the clustering method. These results illustrate the validity of the three eye-tracking metrics (i.e., PTM, RLT, and LFT) in predicting the use of cognitive strategy.^1^

**Figure 9:**
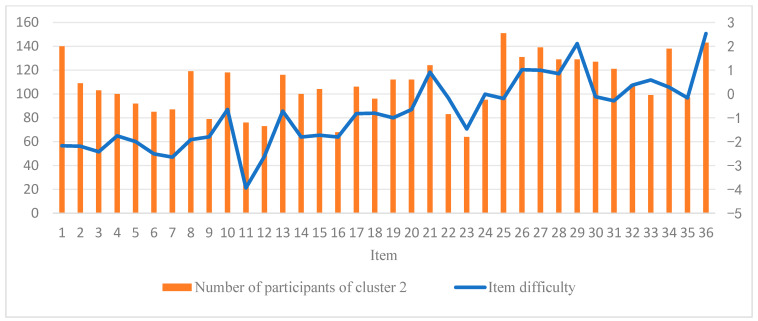
Number of participants of cluster 2 and item difficulty of each item.

**Figure 10:**
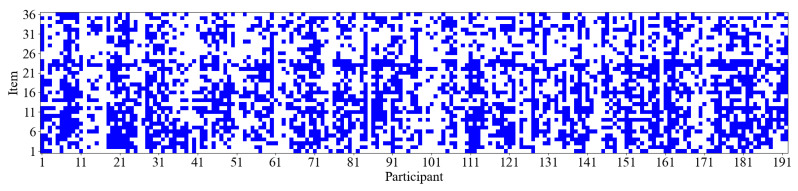
The results of strategy identification for each participant on every item. Blue indicates that the participant used the constructive matching strategy for this item. Conversely, a blank space means the response elimination strategy was used.

Additionally, as illustrated in Figure 6, it appears that participants increasingly adopt the strategy associated with Cluster 2 (i.e., the response elimination strategy) as the test progresses. Figure 9 further displays the item difficulty and the number of participants employing the response elimination strategy for each item. The results suggest a rising trend in item difficulty as item numbers increase, indicating that later items tend to be more challenging. Concurrently, the number of participants using the response elimination strategy also shows an upward trend. The Spearman rank correlation between item difficulty and the number of participants of response elimination strategy usage is 0.58 (*p* < 0.01), suggesting that as item difficulty increases, participants are more likely to employ the response elimination strategy. At the individual level, we further analyzed the relationship between participants’ performance and the frequency of using the constructive matching strategy. The results revealed a significant positive Pearson correlation (r = 0.33, *p* < 0.01) between participants’ scores on the 36 items and the number of times they employed the constructive matching strategy. Specifically, participants who used the constructive matching strategy more frequently tended to achieve higher scores. This finding indicates that the more often an individual uses the constructive matching strategy, the better they perform in the APM. Figure 10 presents the strategy identification results for each participant on every item. This detailed data allows for a meticulous examination of whether participants maintain a consistent cognitive strategy across various items or exhibit variations in their approaches. The results reveal that participants typically employ different strategies for different items, and this is associated with both the item difficulty and their intelligence levels (Liu et al. 2023).

## 5. Conclusions and Discussion

### 5.1. Conclusions

To explore the efficacy of various features in predicting cognitive strategy usage, the present study employed a recursive feature elimination approach in conjunction with a random forest algorithm to identify the most effective predictors. In addition to RA and RT, five key eye-tracking metrics that have been used to study cognitive strategy identification were considered, including PTM, LFT, RLT, NOT, and ROT. The results primarily indicated that PTM, RLT, and LFT are the three most critical features for predicting cognitive strategy usage, with PTM being the most important, followed by RLT and then LFT, while other features played a minor role.

In addition, clustering analysis of the optimal feature subset (including PTM, RLT, and LFT) indicated that the data were optimally categorized into two clusters for the majority of the items. Cluster 1, characterized by higher values of PTM, RLT, and LFT, was inferred to represent the constructive matching strategy. In contrast, Cluster 2, with lower values of these metrics, corresponded to the response elimination strategy. The clustering results showed an 82% consistency with the classifications derived from the MEM, which further illustrates the validity of the three eye-tracking metrics (i.e., PTM, RLT, and LFT) in predicting cognitive strategy usage.

### 5.2. Discussion

The results of the current study both align with and differ from some established findings of previous research. Firstly, the use of eye-tracking metrics such as PTM, LFT, and RLT to predict the cognitive strategy usage corroborates the findings of Vigneau et al. (2006) and Laurence et al. (2018). However, while Vigneau et al. (2006) and Laurence et al. (2018) also suggested NOT and ROT as significant predictors of strategy usage, the present study found their importance to be relatively lower compared to PTM and RLT. This is consistent with the findings of Liu et al. (2023), but it should be noted that their study only considered three eye-tracking matrices (PTM, ROT, and RLT). This discrepancy may arise from differences in methods and the eye-tracking metrics considered. Vigneau et al. (2006) and Laurence et al. (2018) inferred the relationship between eye-tracking metrics and strategy usages by analyzing the relationship between eye-tracking metrics and response outcomes. Raden and Jarosz (2022) affirmed the importance of ROT predictive strategy use by concluding that there was consistency in the strategies used by participants across similar reasoning tasks and that ROT was found to be highly and significantly correlated. Moreover, they did not consider RLT, which was first introduced in Liu et al. (2023). As a result, RLT’s contribution to strategy identification may have been overshadowed by ROT and NOT. Another possibility reason is that ROT is more likely to be used to predict participants’ scores, is correlated with participants’ reasoning ability, and is not an efficient metric for inferring strategy use. In the present study, strategies were directly used as the dependent variable, combined with a general feature selection method, and multiple eye-tracking metrics were compared simultaneously, increasing the reliability of the results.

It is important to note that the five metrics selected for the present study have been demonstrated in prior research to effectively infer the strategies employed by individuals, specifically referring to the constructive matching and response elimination strategies. Based on these well-defined strategies, our study sought to identify the most effective combination of eye-tracking metrics for inferring individual strategies more efficiently. The current study’s findings, that PTM, RLT, and LFT are the best predictors of cognitive strategies, are theoretically reliable. For instance, high PTM, RLT, and LFT values, which indicate that participants spent more time in the matrix interest area and a higher percentage of that time before their first glance at the response options, suggest that individuals primarily use the matrix area for reasoning and constructing mental representations of the correct answers.

Furthermore, the observed trend of increased reliance on response elimination as item difficulty rises is consistent with prior research (e.g., Liu et al. 2023; Gonthier and Roulin 2020). This trend may also help explain why RT were significantly longer for participants using the response elimination strategy compared to those employing the constructive matching strategy. Participants may initially attempt a constructive matching approach on more challenging items; however, when reasoning fails and they cannot mentally represent an answer, they shift to response elimination. This shift results in longer time spent on the item and may lead to participants being classified as using response elimination. Recent findings by Wang and Zhan (2024) further support this, indicating that as item difficulty increases, participants not only spend more time but also demonstrate a higher frequency of strategy shifts within each item.

The present study has some limitations. First, the random forest algorithm used in the present study is a supervised machine learning algorithm, and the strategy labels in the dataset are derived from the MEM. To the best of our knowledge, the MEM is currently the only model that can objectively and quantitatively differentiate between strategies using eye-tracking metrics. While the identification results of this model have some validity and have been used in other supervised machine learning studies (Wang et al. 2024), the labeling may introduce some bias, particularly when used to classify strategies into three categories. Future research could develop new objective and effective methods for distinguishing between strategies, followed by further validation of the current results.

Second, the present study primarily utilized only five eye-tracking metrics, with RLT being introduced and used for the first time in Liu et al. (2023). Although RLT has proven to be effective so far, it has been used in a very limited number of studies. The validity of this metric can be further verified, and its introduction may challenge the results of previous studies. For instance, do ROT and NOT still play important roles when considering RLT? Moreover, future research might consider integrating additional metrics and combining eye-tracking data with other biometric indicators, such as the electroencephalogram. This multimodal approach may provide a more comprehensive understanding of the cognitive processes underlying strategy use. Such a method could help elucidate the complex interactions between cognitive, neural, and behavioral factors in reasoning and strategy usage (Zhu and Lv 2023; Jamal et al. 2023).

It is noteworthy that, based on the PTM, RLT, and LFT metrics, the consistency between the data-driven K-means clustering algorithm and the theoretically driven MEM in the present study was 76%, which is somewhat below expectations and lower than the consistency with the results of Liu et al. (2023)’s study (82%). This discrepancy may stem from two main factors: first, the K-means algorithm is relatively susceptible to outliers, which may lead to the misclassification of certain data points (Olukanmi et al. 2022). Second, the theoretically driven MEM incorporates additional information, such as individual response accuracy, which may enhance its robustness in differentiating cognitive strategies. As a result, under the same set of indicators, the data-driven K-means clustering demonstrated lower consistency with the theoretically driven MEM than anticipated. It is important to note that K-means represents only one type of data-driven approach; future research could explore alternative data-driven algorithms to further evaluate and compare the effectiveness and differences between data-driven and theoretically driven methods in identifying cognitive strategies.

Finally, the study’s sample consisted predominantly of college students, which may limit the generalizability of the findings to broader populations. College students typically have higher cognitive abilities, and their eye-tracking patterns may differ from those of minors or older adults (Niebaum and Munakata 2023; Thibaut et al. 2011; Glady et al. 2016). To enhance the reliability and generalizability of these findings, future studies should consider replicating this research with diverse datasets, including those from Vigneau et al. (2006), Laurence et al. (2018), or other unpublished sources. Such efforts would not only bolster the credibility of the identified eye-tracking metrics—PTM, RLT, and LFT—but also provide more robust evidence for their links to cognitive strategies. Moreover, expanding the sample to include varied age groups, educational backgrounds, and cultural contexts would enrich our understanding of how cognitive strategies differ across populations, thereby broadening the applicability and impact of our conclusions.

## Data Availability

The original contributions presented in the study are included in the article, further inquiries can be directed to the corresponding authors.

## Note

1 A cut-off point of 0.5 was used for MEM, and then compared with the results of the K-means clustering.
